# Comparative Analysis of the Complete Mitochondrial Genomes of *Apium graveolens* and *Apium leptophyllum* Provide Insights into Evolution and Phylogeny Relationships

**DOI:** 10.3390/ijms241914615

**Published:** 2023-09-27

**Authors:** Xiaoyan Li, Mengyao Li, Weilong Li, Jin Zhou, Qiuju Han, Wei Lu, Qin Luo, Shunhua Zhu, Aisheng Xiong, Guofei Tan, Yangxia Zheng

**Affiliations:** 1 College of Horticulture, Sichuan Agricultural University, Chengdu 611130, China; lxy2324804342@163.com (X.L.); limy@sicau.edu.cn (M.L.); 15865899720@163.com (W.L.); jinxyzhou@163.com (J.Z.); hanqiuju0816@163.com (Q.H.); luwei-1213@163.com (W.L.); 2 Institute of Horticulture, Guizhou Academy of Agricultural Sciences, Guiyang 550006, China; lqing985@foxmail.com (Q.L.); zsh2801@163.com (S.Z.); 3 College of Horticulture, Nanjing Agricultural University, Nanjing 611130, China; xiongaisheng@njau.edu.cn

**Keywords:** *Apium graveolens*, *Apium leptophyllum*, mitochondrial genome, SSRs, RNA editing, phylogeny

## Abstract

The genus *Apium,* belonging to the family Apiaceae, comprises roughly 20 species. Only two species, *Apium graveolens* and *Apium leptophyllum,* are available in China and are both rich in nutrients and have favorable medicinal properties. However, the lack of genomic data has severely constrained the study of genetics and evolution in *Apium* plants. In this study, Illumina NovaSeq 6000 and Nanopore sequencing platforms were employed to identify the mitochondrial genomes of *A. graveolens* and *A. leptophyllum*. The complete lengths of the mitochondrial genomes of *A. graveolens* and *A. leptophyllum* were 263,017 bp and 260,164 bp, respectively, and contained 39 and 36 protein-coding genes, five and six rRNA genes, and 19 and 20 tRNA genes. Consistent with most angiosperms, both *A. graveolens* and *A. leptophyllum* showed a preference for codons encoding leucine (Leu). In the mitochondrial genome of *A. graveolens*, 335 SSRs were detected, which is higher than the 196 SSRs found in the mitochondrial genome of *A. leptophyllum*. Studies have shown that the most common RNA editing type is C-to-U, but, in our study, both *A. graveolens* and *A. leptophyllum* exhibited the U-C editing type. Furthermore, the transfer of the mitochondrial genomes of *A. graveolens* and *A. leptophyllum* into the chloroplast genomes revealed homologous sequences, accounting for 8.14% and 4.89% of the mitochondrial genome, respectively. Lastly, in comparing the mitochondrial genomes of 29 species, it was found that *A. graveolens*, *A. leptophyllum*, and *Daucus carota* form a sister group with a support rate of 100%. Overall, this investigation furnishes extensive insights into the mitochondrial genomes of *A. graveolens* and *A. leptophyllum*, thereby enhancing comprehension of the traits and evolutionary patterns within the *Apium* genus. Additionally, it offers supplementary data for evolutionary and comparative genomic analyses of other species within the Apiaceae family.

## 1. Introduction

According to previous studies, plant organelle genomes originated from ancient endosymbiotic bacteria approximately one billion years ago and play a decisive role in various essential life processes such as photosynthesis, cellular respiration, and ATP synthesis [1,2]. Mitochondria, as key organelles for energy metabolism in eukaryotic cells, play an instrumental role in development, reproduction, and various biochemical processes [3]. In addition to synthesizing adenosine triphosphate (ATP) to provide cellular energy [4], they also participate in cell differentiation, signal transduction, and apoptosis [5]. The mitochondrial genome (also known as the mitogenome) has significant gene coding capacity [6]. Unlike the nuclear genome, mitochondria are variable-sized double-stranded DNA molecules, and each cell may contain more than 1000 mitochondrial genomes, known as heteroplasmy [7]. The advent of high-throughput sequencing technologies has greatly facilitated the exploration of animal and plant mitochondrial genomes, revealing the diversity in genome organization, structure, and gene content [8]. Moreover, the high conservation of mitochondrial genomes makes them powerful markers for genetic and phylogenetic research applications [9]. The size of mitochondrial genomes considerably varies among different classes of plants and even among species within the same family [10]. For example, the mitochondrial DNA size of *Citrullus lanatus* is 379 kb [11], while *Cucumis melo* has a mitochondrial genome size of 2740 kb [12]. Recent research has found that plant mitochondrial genomes generally exist in circular, linear, or branched forms, and some exhibit multi-branch structures such as the *Picea sitchens* [13]. These structural variations reflect differences in the amount of repetitive DNA, leading to genome recombination and structural dynamics. Notably, their sizes range from 222 kb in *Brassica napus* to 11.3 Mb in *Silene conica* [14]. The Siberian larch (*Larix sibirica* Ledeb.) mitochondrial genome is currently known to be the largest mitogenome in plants, estimated to have a total size of 11.7 Mb [15].

Plant mitochondrial genomes possess many unique features, with complex and dynamic structures [16]. They contain abundant repetitive sequences, which can lead to rapid genome rearrangements [17]. The utility of mitochondrial genome sequences as genetic markers has been extensively documented, with several mitochondrial genes such as *atp1*, *cob*, and *cox1/2/3* being widely utilized for resolving phylogenetic relationships between lineages and conducting biodiversity analyses [18,19]. Assembling and annotating the mitochondrial genome of the medicinal plant *Bupleurum chinense* revealed that mitochondrial genes are conserved during evolution, providing a foundation for understanding the genetic variation, phylogenetics, and breeding of *B. chinense* [20]. Varré et al. reported that the variation among potato varieties was zero based on the analyses of the potato mitochondrial whole-genome sequence, multi-chromosomal configuration, and transcriptome, while a comparative analysis with other Solanaceae species aided in studying the evolutionary history of its mitochondrial genome [21]. Therefore, investigating mitochondrial genomes has considerable implications for understanding mitochondrial function, and the regulation of cellular metabolism. Moreover, it offers crucial points of reference and theoretical underpinnings for the investigation of species evolution and genetic diversity.

The genus *Apium*, belonging to the family Apiaceae, consists of annual or perennial herbaceous plants distributed in temperate regions [22]. In China, there are only two celery varieties, namely *Apium graveolens* L. and *Apium leptophyllum* (Pers.) F. Muell. The former is one of the most important vegetables in the Apiaceae family and is extensively cultivated worldwide [23]. Apart from being rich in vitamins, phenolic compounds, apigenin, and other nutrients, celery extracts also have medicinal value, such as antibacterial, anti-inflammatory, glucose-lowering, and lipid-lowering properties [24]. Similarly, the latter exerts antibacterial activity against pathogens and fungal strains in its volatile oil, and also exhibits a satisfactory free radical scavenging activity against DPPH(2,2-Diphenyl-1-picrylhydrazyl) [25]. Both of these plants possess desirable medicinal functions and have certain similarities in terms of plant morphology. However, the lack of genetic information and studies on the systematic evolution of the *Apium* genus has hindered the development and utilization of this genus.

Therefore, in this study, the mitochondrial genomes of *A. graveolens* and *A. leptophyllum* were sequenced and assembled. The genome repeats, RNA editing sites, and gene transfer events in the mitochondrial genomes of these two celery species were analyzed and compared with mitochondrial sequences of other Apiaceae plants. Our study provides comprehensive information on the mitochondrial genomes of *A. graveolens* and *A. leptophyllum*, allowing for a better understanding of the characteristics and evolution of the *Apium* genus. Additionally, our research provides additional information for the evolution and comparative genomics studies of other species in the Apiaceae family.

## 2. Results

### 2.1. Analysis of Mitochondrial Genome Assembly and Annotation Results

**Figure 1:**
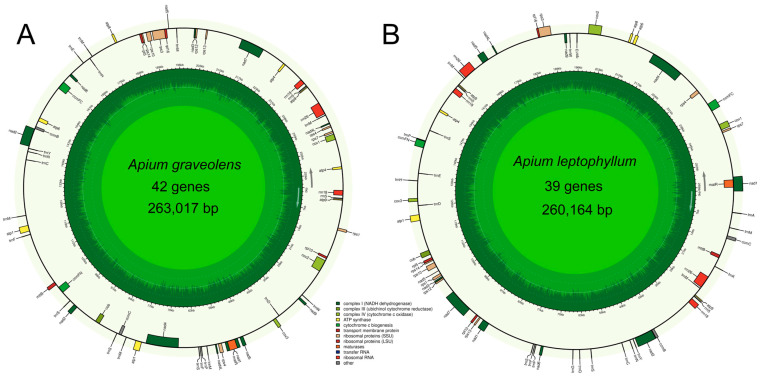
Gene map of the complete mitochondrial genome of *A. graveolens* (**A**) and *A. leptophyllum* (**B**). Genomic characteristics transcribed counter-clockwise are indicated on the inside of the circles. GC content is presented on the inner circle indicated by the dark green plot.

The complete circular mitochondrial genomes of *A. graveolens* and *A. leptophyllum* were obtained by the filtering, assembly, and correction of raw data using the Illumina NovaSeq 6000 sequencing platform. The mitochondrial genome lengths of *A. graveolens* and *A. leptophyllum* were 263,017 bp and 260,164 bp, respectively (Figure 1 and Supplementary Table S1). Likewise, the genome sequences of both species were very similar in length, with GC contents of 45.16% and 45.44% for *A. graveolens* and *A. leptophyllum*, respectively. As anticipated, their base composition showed an AT preference. A total of 63 genes were identified in the mitochondrial genome of *A. graveolens*, including 19 tRNA genes, five rRNA genes, and 39 protein-coding genes. In contrast, *A. leptophyllum* had 62 genes, including 20 tRNA genes, six rRNA genes, and 36 protein-coding genes (Supplementary Table S2). Comparing the annotated genomes exposed significant differences in the genes of both species. For instance, *A. graveolens* contained four additional duplicated protein-coding genes (*atp1*, *atp4*, *nad4L*, and *rps4*) and three duplicated *trnM-CAT* genes compared to *A. leptophyllum*. Meanwhile, *A. leptophyllum* had five unique tRNA genes (*trnA-TGC*, *trnG-GCC*, *trnK-TTT*, *trnP-CGG*, and *trnQ-TTG*) (Supplementary Table S3). Interestingly, the AT-skew and GC-skew values in the complete genome of *A. graveolens* were negative, whereas they were positive in *A. leptophyllum*. Additionally, the AT-skew values of protein-coding genes and tRNA in both *A. graveolens* and *A. leptophyllum* were negative, whereas the GC-skew values were positive. Lastly, the AT-skew values of the complete genome and rRNA were positive, whilst the AT-skew values of protein-coding genes and tRNA were negative (Supplementary Table S4).

### 2.2. RSCU Analysis

**Figure 2:**
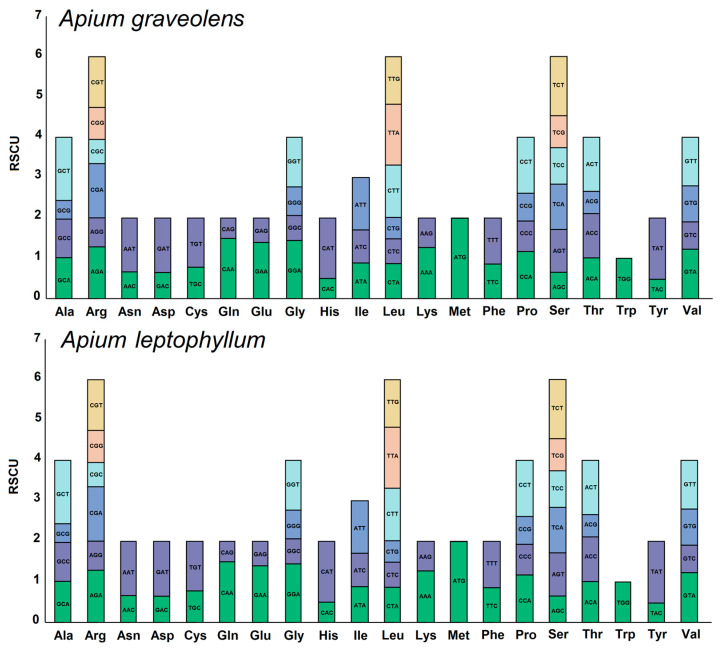
Relative synonymous codon usage (RSCU) of mitochondrial genomes of *A. graveolens* and *A. leptophyllum*.

Codon usage bias analysis of the mitochondria of *A. graveolens* and *A. leptophyllum* revealed consistent usage patterns of codons for different amino acids. The usage patterns are detailed in Figure 2 and Supplementary Table S5. In the mitochondrial genomes of *A. graveolens* and *A. leptophyllum*, 9050 and 9338 codons were detected, respectively. The usage frequency of the leucine (Leu) codon was highest in both genomes, with 964 and 955 occurrences for *A. graveolens* and *A. leptophyllum*, respectively. Serine (Ser) codons had the next highest frequency, with 880 and 885 occurrences. This phenomenon is consistent with most species. Furthermore, the analysis determined that among the codons in the mitochondrial protein-coding genes (PCGs), excluding the start codon methionine (AUG) with an RSCU (Relative synonymous codon usage) value of one, a total of 31 codons in *A. graveolens* and 32 codons in *A. leptophyllum* had an RSCU value greater than one, indicating a general preference for codon usage in mitochondrial PCGs. Notably, methionine (Met) showed a high preference for the AUG codon, with the highest RSCU value of 1.99 in *A. graveolens* and *A. leptophyllum*. Termination codon (Ter) UAA also exhibited a higher preference, with RSCU values of 1.78 in *A. graveolens* and 1.68 in *A. leptophyllum*. It is worthwhile emphasizing that phenylalanine (Phe) had a maximum RSCU value lower than 1.2, indicating a weaker codon usage preference. Among the 31 codons in *A. graveolens* and the 32 codons in *A. leptophyllum* with RSCU values greater than one, only three codons (CUC, UUG, and AUG) ended with G/C, whereas the remaining ended with A/T bases, accounting for 90.3% and 90.6% of codons in *A. graveolens* and *A. leptophyllum*, respectively. This observation implies that the mitochondrial genome codons in *A. graveolens* and *A. leptophyllum* are more inclined to end with A/T bases than G/C.

### 2.3. SSRs and Repeat Sequence Analysis

**Figure 3:**
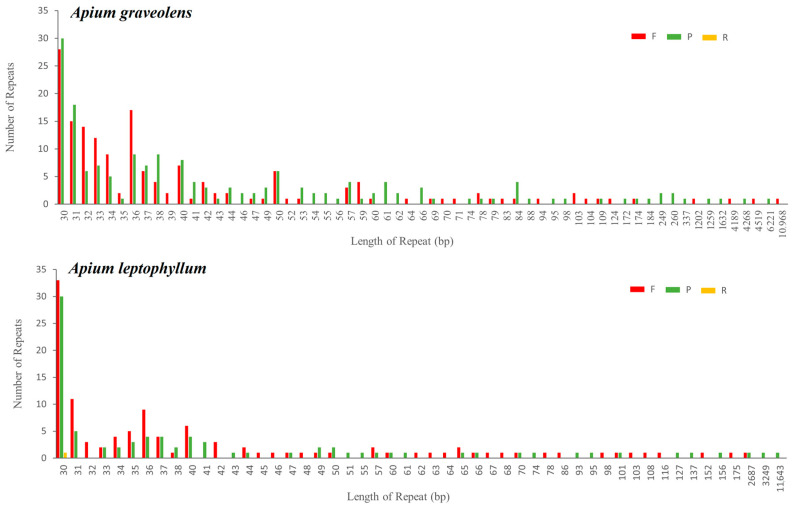
Number of repeat sequences in *A. graveolens* and *A. leptophyllum* mitochondrial genomes. The horizontal coordinate is the type of scattered repeat sequences and the vertical coordinate is the number of scattered repeat sequences. F: forward repeats; P: palindromic repeats: R: reverse repeats.

A total of 335 and 196 simple sequence repeats (SSRs) were detected in the mitochondrial genomes of *A. graveolens* and *A. leptophyllum*, respectively (with lengths greater than or equal to 30 bp). Among them, 163 and 111 were forward repeats, and 172 and 84 were palindromic repeats. Additionally, there was one reverse repeat in the mitochondrial genome of *A. leptophyllum* but no complementary repeats in either species (Figure 3). The number of repeats in the mitochondrial genomes of *A. graveolens* and *A. leptophyllum* accounted for 18.90% and 11.02% of their respective genomes. The longest repeat sequence in *A. graveolens* was a forward repeat with a length of 10,968 bp, while that in *A. leptophyllum* was a palindromic repeat with a length of 11,643 bp. The majority of repeat sequences in both species (96.11–97.95%) had lengths ranging from 30 to 199 bp. Nevertheless, there were eight repeat sequences longer than 1000 bp in *A. graveolens* and four in *A. leptophyllum*, accounting for 13.03% and 7.79% of their respective genomes (Supplementary Tables S6 and S7).

### 2.4. The Prediction of RNA Editing

**Figure 4:**
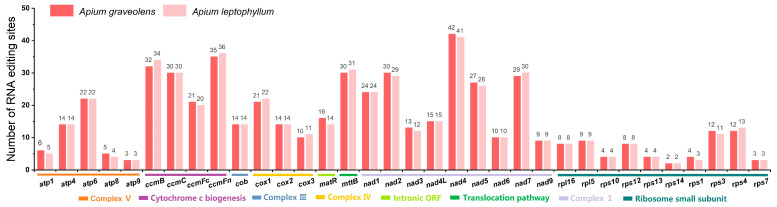
RNA editing sites in different coding genes of *A. graveolens* and *A. leptophyllum*.

RNA editing site analysis was performed on the two celery species, *A. graveolens* and *A. leptophyllum*, of which 538 and 535 RNA editing sites were identified in 35 gene types, respectively. Among them, the *nad4* gene was found to have the highest number of predicted RNA editing sites, with 42 in *A. graveolens* and 41 in *A. leptophyllum* (Figure 4). Current research suggests that the most prevalent type of RNA editing is C-to-U, although, in some species, U-to-C editing can also be observed. Interestingly, both *A. graveolens* and *A. leptophyllum* showed U-to-C editing, with 20 and 27 instances, respectively (Supplementary Table S8). Moreover, there were 24 common substitution patterns of RNA editing between the two species. Among these substitutions, the most frequent amino acid changes were serine-to-leucine (S-to-L), proline-to-leucine (P-to-L), and serine-to-phenylalanine (S-to-F), whereas serine-to-proline (S-to-P) was the least common. Ribosomal proteins (*rps1*, *rps7*, *rps13*, *rps14*, and *rps16*) and ATPase subunit (*atp8*) had relatively fewer derived RNA editing substitutions (1–8 sites), whereas other genes had 12–95 editing sites. Additionally, the *nad1*, *nad2*, and *nad5* genes contained both cis- and trans-spliced introns, whereas the *ccmFC*, *nad4*, *nad7*, *rps3*, *rps10*, and *rps14* genes only encompassed trans-spliced introns, indicating significant editing in NADH dehydrogenase subunit transcripts.

### 2.5. Structural Comparison of A. graveolens and A. leptophyllum

**Figure 5:**
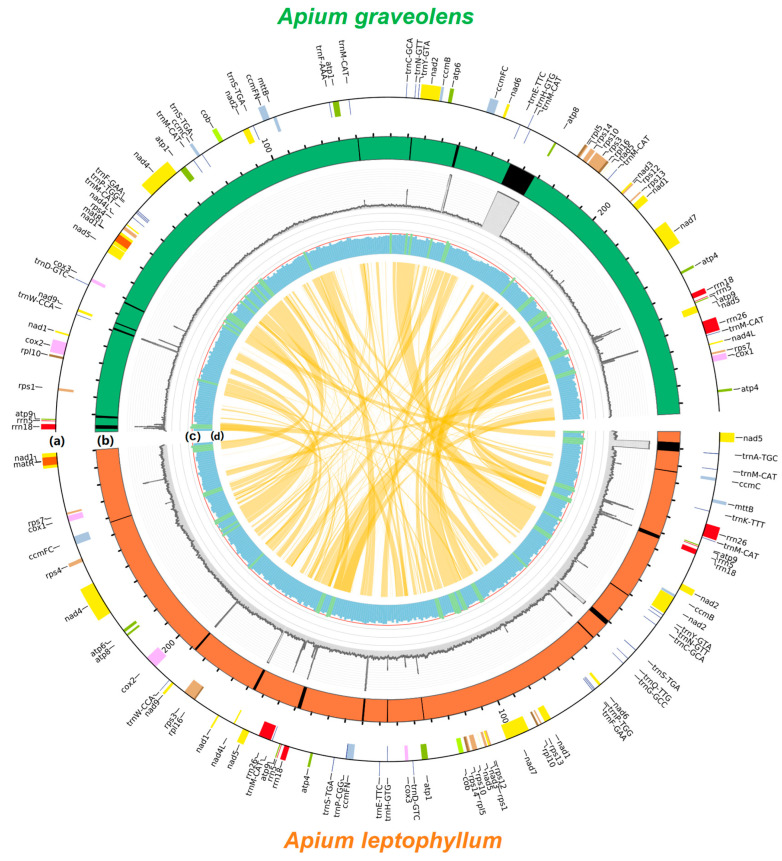
Schematic representation of homologous sequences between *A. graveolens* and *A. leptophyllum* mitogenomes. (**a**) Gene blocks shown on the outside and inside the circle were transcribed clockwise and counter-clockwise, respectively. Genes from the same complex are similarly colored. (**b**) Plastid-derived fragments characterized by the black blocks inlaid in the karyotypes. (**c**) The GC content in 1000 bp windows. (**d**) The orange-colored band in the center show links between syntenic blocks among the two mitogenomes.

Owing to the relatively large size of the mitochondrial genome, a protein-coding region was extracted from the *rrn18* of *A. graveolens* and the *nad5* of *A. leptophyllum* to generate a new circular map. This sequence comprised 68 genes, of which 48 were protein-coding genes. As illustrated in Figure 5, there were 21 plastid-derived fragments in the mitochondrial genome of *A. graveolens*, fewer than the 27 fragments in *A. leptophyllum*, insinuating a faster turnover of DNA in *A. leptophyllum* compared to *A. graveolens*. Aside from this, the mitochondrial genomes of both species exhibited extremely high homology in their sequence structure.

### 2.6. Sequence Similarity between Mitochondrial and Chloroplast Genomes

**Figure 6:**
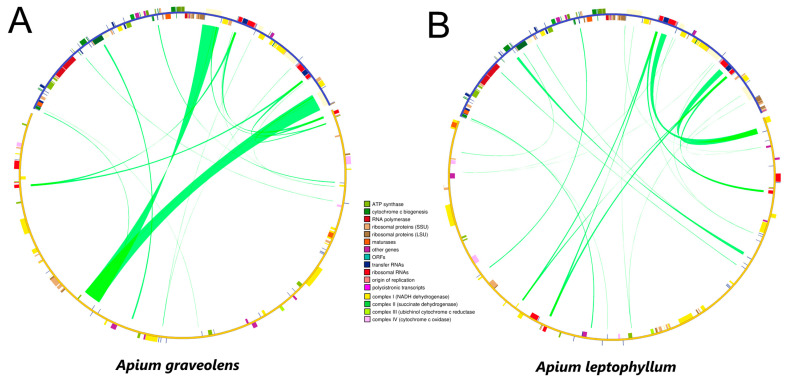
Schematic for the chloroplast-to-mitochondrial gene transfer in *A. graveolens* and *A. leptophyllum*. (**A**) Sequence similarity between the mitochondrial and chloroplast genomes in *A. graveolens*. (**B**) Sequence similarity between the mitochondrial and chloroplast genomes in *A. leptophyllum*. Note: The sequences above represent chloroplast sequences, while the sequences below represent mitochondrial sequences. Chloroplast coding sequences (CDS) are shown in green, while mitochondrial CDS are shown in red. Yellow indicates rRNA, blue represents tRNA, and gray represents introns. The green lines connecting regions indicate homologous sequences.

Sequence similarity analysis determined that the identified homologous sequences in the mitochondrial and chloroplast genomes of *A. graveolens* and *A. leptophyllum* were 21,435 bp and 12,739 bp, respectively, accounting for 8.14% and 4.89% of the mitochondrial genome (Figure 6). In both celery species, 23 plastid-derived genome fragments, including genes and intergenic regions, were detected, with 12 fragments ranging from 152 to 7488 bp. Furthermore, among the genome fragments with over 93% sequence homology to the original chloroplast, 17 fragments were present in *A. graveolens* and 15 fragments in *A. leptophyllum*. Moreover, it was found that *psbA* in *A. graveolens* and *trnH-GUG* in *A. leptophyllum* had 100% sequence homology. There were also 11 complete chloroplast genes (*ndhB, rps7, rps12, ycf2, rpl23, atpe, atpB, rbcL, psbB, pet1,* and *petg*), six tRNAs (*trnL-caa*, *trnV-gac*, *tRNA-Ile*, *trnM-cau*, *trnW-cca*, and *trnP-UgG*), and one rRNA (*Rrn16*). Finally, nucleotide substitutions were observed in tRNAs, attributable to plastid copy.

### 2.7. The Ka/Ks Ratio and Nucleotide Diversity (Pi) Values

**Figure 7:**
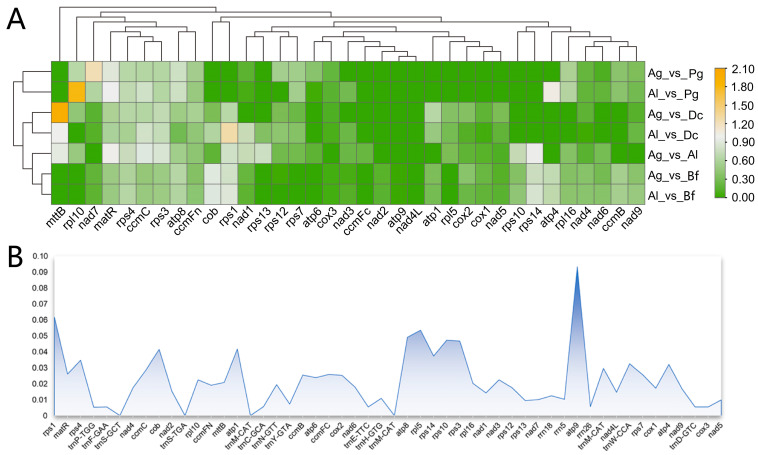
KaKs substitution rates and Pi values of genes. (**A**) Analysis of KaKs substitution rates among five Apiaceae species. Ag: *A. graveolens*; Al: *A. leptophyllum*; Pg: *P. ginseng*; Dc: *D. carota*; Bf: *B. falcatum*. (**B**) Nucleotide diversity (Pi) values among *A. graveolens* and *A. leptophyllum*.

According to Figure 7A, three Apiaceae plants, ginseng, carrot, and Bupleurum, manifested certain variations in protein-coding genes compared to *A. graveolens* and *A. leptophyllum*, with all genes having a Ka/Ks value less than 0.5 (Supplementary Table S9). This finding signaled that these genes are highly conserved during plant evolution and are subject to purifying selection pressure. In *A. leptophyllum* vs. *Panax ginseng* (KF735063.1) and *A. leptophyllum* vs. *Daucus carota* (JQ248574.1), two genes (*atp4*, *rpl10*, and *mttB*, *rps1*) had a Ka/Ks value greater than one, whilst no genes had a Ka/Ks value greater than one in *A. graveolens* vs. *B. falcatum* (KX887330.1) and *A. leptophyllum* vs. *B. falcatum*, inferring that these genes have undergone significant purifying selection. Furthermore, in comparison to three other plants, *A. graveolens* and *A. leptophyllum* had Ka/Ks values less than one for *nad1*, *nad2*, *nad3*, and *nad4*, suggesting positive selection in these four genes among different plants in the Apiaceae family.

The calculation of the Pi values for the 52 shared genes between *A. graveolens* and *A. leptophyllum* uncovered that the variation for 47 genes ranged between 0.00533 and 0.06164 (Figure 7B). More importantly, the gene with the highest variation level was *atp9*, with a Pi value of 0.09333, followed by *rps1* and *rpl5,* with Pi values of 0.06164 and 0.5344, respectively. Among the detected variant genes, 35 were protein-coding genes, 10 were tRNA genes, and three were rRNA genes. Indeed, the three genes with the highest variation level were all protein-coding genes, signifying that these three hotspots likely contain information on evolutionary sites and could be potential molecular markers. Collectively, these results indicate that nucleotide sequence variation chiefly occurs in the coding regions (CDs) of protein-coding genes, which may be the primary cause of variation among Apiaceae plants.

### 2.8. Comparative Genomics Analysis

**Figure 8:**
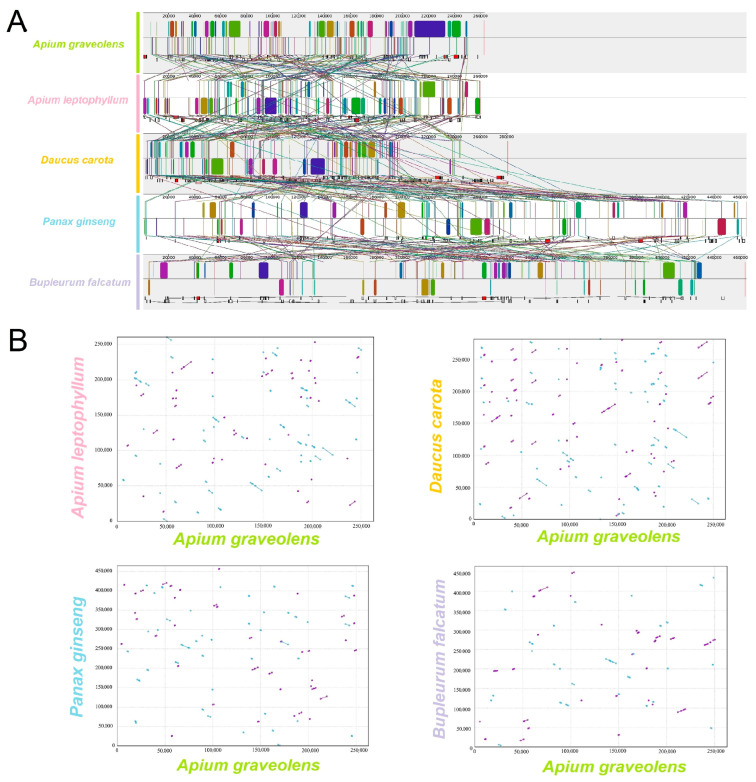
Collinearity analysis of the mitochondrial genome of Apiaceae. (**A**) Analysis of covariance between the mitochondrial sequences of *A. graveolens*, *A. leptophyllum*, and near-origin species. The long squares in the figure represent the similarity between genomes, and the connecting lines between the long squares represent the covariance relationship. The short squares denote the gene positions of each genome. Additionally, white represents CDS, green represents tRNA, and red represents rRNA. (**B**) Dot plot of two celery species with close relatives. Horizontal coordinates in each box represent assembly sequences, vertical coordinates represent other sequences, purple lines in the boxes represent forward comparisons, and blue lines represent reverse complementary comparisons.

Using the mitochondrial genome sequence of celery as a reference, a comparative analysis of the mitochondrial genomes of *A. graveolens*, *A. leptophyllum*, and three other Apiaceae plants was conducted to assess their co-linearity. The results exposed significant gene rearrangements and complex structural variations among the five plant mitochondrial genomes (Figure 8A,B). *A. graveolens* and *A. leptophyllum* possessed similar genome sizes but exhibited substantial structural differences, indicating a weak co-linearity between them. At the same time, *D. carota* had a comparable mitochondrial genome size and structure to *A. graveolens*, displaying some degree of co-linearity. Compared to the remaining three Apiaceae plants, *A. graveolens* and *A. leptophyllum* had smaller mitochondrial genomes and relatively shorter homologous regions, suggesting a close correlation between the degree of variation among Apiaceae species and mitochondrial genome size.

### 2.9. Phylogenetic Relationship

**Figure 9:**
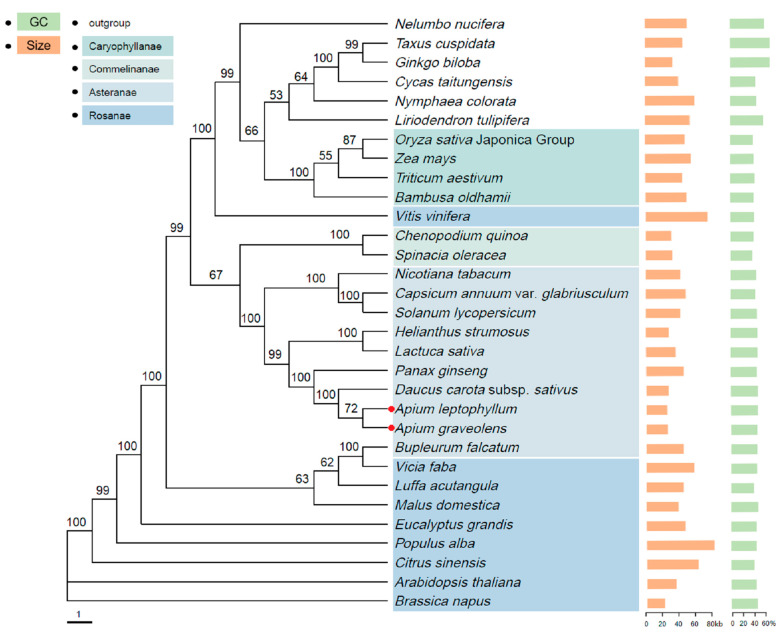
Maximum likelihood (ML) phylogenetic tree based on 31 species. Four colored lines represent four families and their phylogenetic relationships, namely *Caryophyllance*, *Commelinanae*, *Asteranae,* and *Rosanae*. The remaining species had no color and were classified as outgroups. The green and orange colors on the right represent GC content and mitogenome size, respectively. Numbers beside nodes indicate bootstrap support values.

Based on the annotation of the mitochondrial genomes of *A. graveolens* and *A. leptophyllum*, a phylogenetic tree was constructed using maximum likelihood analysis, with *Nelumbo nucifera* (KR610474.1), *Liriodendron tulipifera* (MK340747.1), *Nymphaea colorata* (KY889142.1), *Taxus cuspidata* (MN593023.1), *Ginkgo biloba* (KM672373.1), and *Cycas taitungensis* (AP009381.1) as outgroups, whilst the mitochondrial genome data of 29 other plant species were downloaded from NCBI. As depicted in Figure 9, *A. graveolens* and *A. leptophyllum* formed a branch within *Asteranae*, with marginal differences in mitochondrial genome size and GC content, supported by a node support value of 72%. Additionally, the two celery species are sister groups to *Daucus carota*, forming a highly supported clade with 100% support. Furthermore, *A. graveolens*, *A. leptophyllum*, *Daucus carota*, *Panax ginseng*, *Lactuca sativa*, and *Helianthus strumosus* clustered together within *Rosanae*, indicating moderate relatedness. More importantly, the mitochondrial genome sizes within *Rosanae* significantly differed from those of the two *Apium* species, implying substantial differences between *Rosanae* and the two celery species.

## 3. Discussion

Herein, the mitochondrial genomes of *A. graveolens* and *A. leptophyllum* were assembled using second- and third-generation high-throughput sequencing technologies. The results revealed that the genome sequence lengths and GC contents of the two plants were very similar. The mitochondrial genome of *A. graveolens* had a length of 263,017 bp and a GC content of 45.16%. Similarly, the mitochondrial genome of *A. leptophyllum* had a length of 260,164 bp and a GC content of 45.44%. These values were comparable to the mitochondrial genomes of other sequenced plants such as *C.duntsa* (45.62%) [26], *B. chinense* (45.68%) [20], and *B. juncea* (45.24%) [27]. Nonetheless, significant differences were noted in the identified genes between the two species. To begin, *A. graveolens* had seven additional genes (*atp1*, *atp4*, *nad4L*, *rps4*, and 3 *trnM-CAT*) compared to *A. leptophyllum*. However, *A. leptophyllum* contained five unique tRNA genes (*trnA-TGC*, *trnG-GCC*, *trnK-TTT*, *trnP-CGG*, and *trnQ-TTG*). Therefore, it can be deduced that at the whole genome level, the mitochondrial genome size, structure, number, and types of *A. graveolens* and *A. leptophyllum*, as well as their base composition and GC content, are highly conserved. Most mitochondrial variations occur in the intergenic regions, which is consistent with the findings from the grape mitochondrial genome study [14].

Throughout the course of plant evolution, the mitochondrial genome undergoes a multitude of modifications, including alterations in genome structure and nucleotide composition, as well as the loss and transfer of protein-coding and tRNA genes [28]. This preference is considered the result of a combination of natural selection, species mutation, and genetic drift [29]. Therefore, exploring the characteristics and fluctuations in codon usage strategies can assist in the analysis of the phylogenetics and evolutionary process of the mitochondrial genome [30]. The codon preference analysis results showed that, in line with most plant species, both *A. graveolens* and *A. leptophyllum* had the highest frequency of leucine (Leu) codons, with 964 and 955 occurrences, respectively [31]. Additionally, the research also revealed that dicot plants exhibit a bias towards A/T-ending codons [32]. In the protein-coding genes of *A. graveolens* and *A. leptophyllum*, a strong A/T bias was observed in the preferred codons with RSCU values greater than one. RNA editing is closely associated with the potential molecular functions and physiological processes of mitochondria in higher plants [33]. Thus, investigating RNA editing sites yields a better understanding of the expression of mitochondrial and chloroplast genes in plants. In our study, 544 and 520 RNA editing sites were predicted in *A. graveolens* and *A. leptophyllum*, slightly higher than those predicted in other plants such as *Arabidopsis* (441 sites) [34] and rice (491 sites) [35].

Genomic repetitive sequences are abundant in mitotic genomes and serve as important evidence for assessing species evolution and genetic characteristics [36]. Simple sequence repeats (SSRs) can be used to identify different types of genomes and environmental characteristics of species, providing a scientific basis for species genetics [37]. The mitochondrial genomes of *A. graveolens* and *A. leptophyllum* contain 335 and 196 pairs of repetitive sequences (length ≥ 30 bp), accounting for 18.90% and 11.02% of the two mitochondrial genomes, respectively, and no complementary repetitive sequences were detected. However, the differences in the quantity and types of repeats may be caused by gene duplication or variation, as well as geographical and ecological factors [38]. To further analyze the degree of evolution among species, a Ka/Ks analysis was carried out on the mitochondrial genes of three other Apiaceae plants. The results indicate that, compared to *A. graveolens* and *A. leptophyllum*, the protein-coding genes of the other three Apiaceae plants have undergone some degree of variation. The Ka/Ks values for all genes are less than 0.5, indicating that these coding genes are highly conserved and have not undergone rapid evolution during plant evolution [39].

During the evolutionary process of mitochondria, there is a frequent occurrence of DNA transfer events between the mitochondrial genome and the nuclear genome, as well as between different species [40]. Some chloroplast fragments migrate to the mitotic genome, and the length and sequence similarity of the migrated fragments vary among species [41]. In our study, the total lengths of the chloroplast genome transferred to the mitochondrial genome in *A. graveolens* and *A. leptophyllum* were 21,435 bp and 12,739 bp, accounting for 8.14% and 4.89% of the mitochondrial genome, respectively, with tRNA gene transfer being the most common [42]. With rapid advances in sequencing technologies, mitochondrial genome sequencing has become a fundamental approach to solving phylogenetic relationships [43]. In this study, the phylogenetic analysis based on mitochondrial genome sequences of 29 angiosperms demonstrated that *A. graveolens* and *A. leptophyllum* clustered together, signifying highly conserved mitochondrial genomes between them, with the differences potentially originating from intra-species evolution. In summary, the analysis of the mitochondrial genomes of *A. graveolens* and *A. leptophyllum* is anticipated to provide novel insights and evidence for studies on phylogenetics, evolution, and conservation genetics of the *Apium* species.

## 4. Materials and Methods

### 4.1. Materials, DNA Extraction, and Sequencing

The *A. graveolens* cultivar of ‘Jinnan Shiqin’ and the wild seeds of *A. leptophyllum* were obtained from the Modern Agricultural Base of Sichuan Agricultural University, located at coordinates 103°37′39″–103°40′5″ E and 30°32′21″–30°34′35″ N. The seedlings were grown in pots within a controlled-environment growth chamber set at a temperature of 20 °C and a relative humidity of 80%. After two months, the seedlings of *A. graveolens* and *A. leptophyllum* were harvested and quickly frozen in liquid nitrogen. Plant DNA extraction kits (TransGene, Beijing, China) were used to extract genomic DNA from *A. graveolens* and *A. leptophyllum*, and the quality of DNA was checked by using NanoDrop ND 2000 (ThermoFischer, Waltham, MA, USA). The extracted DNA was sheared into fragments using an ultrasonic crusher, and two 150 bp paired-end libraries were prepared from two DNA samples. The sequencing was performed at Genepioneer Biotechnologies (Nanjing, China) using two sequencing strategies Illumina novaseq6000 and Nanopore with paired-end reads of 150 bp.

### 4.2. Mitochondrial Genome Assembly

The raw reads generated from second-generation sequencing were deduplicated and quality-filtered using Fastp software (version 0.20.0, https://github.com/OpenGene/fastp, accessed on 5 October 2022). Canu [R] was used to assemble the three generations of sequencing data into contigs using the following parameters: genomeSize = 5 m and correctedErrorRate = 0.03. Then, contigs were aligned to other completely assembled mitochondrial genomes by blast v2.6 (https://blast.ncbi.nlm.nih.gov/Blast.cgi, accessed on 10 October 2022) and further assembled by NextPolish (version 1.3.1, https://github.com/Nextomics/NextPolish, accessed on 10 October 2022) [44]. Finally, Pilon was used to correct the assembly sequences. The complete mitochondrial genomes of *Apium graveolens* and *Apium leptophyllum* were submitted into the NCBI database with the accession numbers MZ328722 and MZ328723, respectively.

### 4.3. Genome Annotations and Analysis

Protein-coding genes and rRNA were annotated by their similarity to published plant mitochondrial sequences and by using BLAST searches. The tRNA genes were annotated using tRNAscanSE (http://lowelab.ucsc.edu/tRNAscan-SE/, accessed on 20 October 2022) [13]. ORFs were predicted using the NCBI Open Reading Frame Finder (https://www.ncbi.nlm.nih.gov/orffinder/, accessed on 20 October 2022) with the minimum ORF length set at 100 bp. The RNA editing sites (C-to-U) in protein-coding genes were predicted using the online program PREPACT (v3.12.0, http://www.prepact.de/prepact-main.php, accessed on 21 October 2022). The circular map of the mitochondrial genome was constructed using OGDRAW (v1.3.1, https://chlorobox.mpimp-golm.mpg.de/OGDraw.html, accessed on 21 October 2022) [45].

### 4.4. RNA Editing Analysis and Characteristic Analysis of Mitochondrial Genome

The intron splicing pattern, RNA editing site map, and RNA editing sites were predicted using the http://prep.unl.edu/ (accessed on 25 October 2022) method. The protein coding genes of the mitochondrial genome were analyzed for codon preference using Mega 7.0 (v11.0.10) software, and RSCU values were calculated. Interspersed repetitive sequences (IRSs) across the mitochondrial genome were determined by REPuter (https://bibiserv.cebitec.uni-bielefeld.de/reputer/, accessed on 27 October 2022) with the minimum repeat size set to 30 and the hamming distance set to 3. There were four identification forms (forward, palindromic, reverse, complement) for IRS. A BLAST search (https://blast.ncbi.nlm.nih.gov/Blast.cgi, accessed on 27 October 2022) program was used to find the homologous sequences between the chloroplast genome and mitochondrial genome; the similarity was set to 70%, and the E-value was set to 10 × 10^−5^. Homologous sequence results were visualized using Circos (v0.69-5, http://circos.ca/software/download/, accessed on 27 October 2022). The chloroplast genomes of *A. graveolens* and *A. leptophyllum* were downloaded from NCBI with accession numbers MZ328720 and MZ328721, respectively.

### 4.5. Comparative Analysis of Mitochondrial Genome

Sequences of six species from the Apiaceae family, including *Daucus carota* (JQ248574.1), *Bupleurum falcatum* (KX887330.1), *Panax ginseng* (KF735063.1), and *Dendropanax morbifer* (MW073906.1), with their mitochondrial genome, were selected for comparative mitogenome analysis. The MAFFT software (v7.310, https://mafft.cbrc.jp/alignment/software/, accessed on 3 November 2022) was used for multiple nucleotide sequence alignment, and the slide window analysis was subsequently carried out using DnaSP (v5.0, http://www.ub.edu/dnasp, accessed on 3 November 2022) to determine the Pi value. KaKs_Calculator (v2.0, https://sourceforge.net/projects/kakscalculator2/, accessed on 6 November 2022) was used to calculate the values of the nonsynonymous substitution rate (Ka) and synonymous substitution rate (Ks). The genomic alignment was performed by the Mauve software (http://darlinglab.org/mauve, accessed on 10 November 2022) using default parameters, and whole genome alignment visualization was created using CGVIEW (https://www.bioinformatics.org/cgview/download.html, accessed on 3 November 2022) [46].

### 4.6. Phylogenetic Analysis

To investigate the mitochondrial genome evolutionary relationship, 28 completely sequenced mitochondrial genomes of plants, including *Malus domestica* (FR714868.1), *Luffa acutangula* (MT374097.1), *Vicia faba* (KC189947.1), *Populus alba* (MK034705.1), *Citrus sinensis* (MG736621.1), *Brassica napus* (AP006444.1), *Arabidopsis thaliana* (Y08501.2), *Eucalyptus grandis* (MG925370.1), *Vitis vinifera* (FM179380.1), *Solanum lycopersicum* (MF034193.1), *Nicotiana tabacum* (KR780036.1), *Capsicum annuum* (MN196478.1), *Daucus carota* (JQ248574.1), *Bupleurum falcatum* (KX887330.1), *Panax ginseng* (KF735063.1), *Dendropanax morbifer* (MW073906.1), *Lactuca sativa* (MK642355.1), *Helianthus strumosus* (MT588181.1), *Spinacia oleracea* (KY768855.1), *Liriodendron tulipifera* (MK340747.1), *Chenopodium quinoa* (MK182703.1), *Oryza sativa* (BA000029.3), *Zea mays* (AY506529.1), *Triticum aestivum* (AP008982.1), *Bambusa oldhamii* (EU365401.1), *Taxus cuspidata* (MN593023.1), *Cycas taitungensis* (AP009381.1), *Ginkgo biloba* (KM672373.1), *Nymphaea colorata* (KY889142.1), and *Nelumbo nucifera* (KR610474.1), were downloaded from the NCBI (accessed on 25 November 2022). Phylogenetic analysis was performed based on all mitochondrial gene sequences among two *Apium* plants and another 28 species. Sequence alignments were conducted by MAFFT (v7.427) and a maximum-likelihood phylogenetic tree was constructed using RAxML (v8.2.10) with 1000 bootstraps.

## 5. Conclusions

Plant mitochondrial genomes have highly conserved gene content and a relatively slow evolution rate, while their genomic structure, size, and repetitive sequences tend to be variable. Herein, the mitochondrial genomes of *A. graveolens* and *A. leptophyllum* were assembled and annotated, and the annotated genes were analyzed. The total lengths of the *A. graveolens* and *A. leptophyllum* genomes were 263,017 bp and 260,164 bp, with GC contents of 45.16% and 45.44%, respectively, and both showed a preference for AT base composition. Additionally, the usage of codons, repetitive sequences, genome recombination, chloroplast-to-mitochondria DNA transformation, and RNA editing sites was analyzed. Moreover, the phylogenetic tree based on mitochondrial genomes of 29 angiosperms contributed to the scientific classification of *A. graveolens* and *A. leptophyllum*. Our study not only provides information on the genetic characteristics, phylogenetic relationships, and evolution of *A. graveolens* and *A. leptophyllum*, but also serves as an important resource for future investigations into the evolution of mitochondrial genomes in the Apiaceae species.

## Data Availability

The mitochondrial genome sequences of *Apium graveolens* and *Apium leptophyllum* that were generated are deposited at NCBI under the accession numbers MZ328722 and MZ328723, respectively.

## Supplementary Materials

The following supporting information can be downloaded at https://www.mdpi.com/article/10.3390/ijms241914615/s1.
